# Supplementary material for: Locally Recurrent Rectal Cancer According to a Standardized MRI Classification System: A Systematic Review of the Literature
Source: J Clin Med. 2022 Jun 18;11(12):3511. doi: 10.3390/jcm11123511 (PMC9224654; doi:10.3390/jcm11123511)

Supplementary Table S1. Patient demographics and treatment information

Study	Country	Years of study	Type of cohort study	Recurrences only included in study Y/N	Mean or median age (years) (Total population (TP) or recurrences (R))	No. of patients followed / included in study	Male : Female (TP or R)	Stage of primary tumours (TP or R)	Lymph node status (TP or R)	Perioperative treatment of primary tumour (TP or R)	No. of local recurrences (included / classified)	Method of diagnosis of recurrence	Classification system used	Operation performed for primary cancer leading to recurrence (TP or R)
Adachi et al. 1999 [18]	Japan	11	Retrospective	N	(R):<br>61.9	135	(R):<br>4M : 5F	Depth of invasion (R):<br>Shallow part of adventitia = 5<br>Submucosa = 1<br>Muscularis propria = 3	(R):<br>N-ve = 2<br>N+ve = 7	Not reported	21 (10)	Clinical and radiological	Anatomical	(R):<br>Pull-through = 2<br>LAR = 5<br>APER = 2
Adloff M et al. 1985 [19]	France	7	Prospective	N	(R):<br>66	113	(R):<br>10M : 26F	Dukes stage (R):<br>B = 16<br>C = 20	(R):<br>N-ve = 21<br>N+ve = 15	Not reported	36 (36)	Clinical/radiological/histopathological	Anatomical	(R):<br>APER = 36
Baek JY et al. 2019 [20]	Seoul	9	Retrospective	N	(TP):<br>67	122	(TP):<br>67M : 55F	(R):<br>pT3 = 6	(R):<br>pN0 = 6	(R):<br>No neoadjuvant/adjuvant treatment = 6	6 (6)	Clinical/radiological/histopathological	Regional anatomical	(TP):<br>APER: = 2<br>LAR: 120
Beppu N et al. 2017 [21]	Japan	12	Retrospective	N	(TP):<br>63.5	119	(TP):<br>84M :35F	TNM (R):<br>T3 = 9	(TP):<br>N -ve = 44<br>N+ve = 75	(R):<br>Neoadjuvant radiotherapy = 9	9 (9)	Clinical/radiological/histopathological	Regional anatomical	(R):<br>Intersphincteric resection = 6<br>APER = 3
Beppu N et al. 2018 [22]	Japan	13	Retrospective	N	(TP):<br>62	216	(TP):<br>144M : 72F	(R):<br>T3 = 16	(TP):<br>N-ve = 156<br>N+ve = 60	(R):<br>Neoadjuvant short-course radiotherapy = 16	16 (16)	Clinical/biochemical/radiological (confirmed with MRI/PET-CT)	Regional anatomical	(TP):<br>**All TME:**<br>Double-stapling technique = 94<br>Intersphincteric resection = 106<br>APER = 14<br>Hartmann's = 2<br>Additional PSD = 13
Bikhchandani J et. al 2015 [23]	USA	16	Retrospective	Y	(R):<br>66	27	(R):<br>9M : 18F	TNM (R):<br>Tx = 2<br>Tis = 2<br>T1 = 16<br>T2 =7	(R):<br>N0 = 7<br>Unknown = 20	Not reported	32 (32)	EUS/MRI	Anatomical	(R):<br>LE = 27
Bird T et al. 2018 [24]	Australia	19	Prospective	Y	(R):<br>63	98	(R):<br>61M : 37F	TNM (R):<br>T1-T2 N0 = 14,<br>T3N0 = 26,<br>T2N+ve = 2,<br>T3N+ve = 21,<br>T4N0 = 9,<br>T4N+ = 6,<br>Unknown = 20	(R):<br>N-ve = 49<br>N+ve = 29<br>Unknown = 20	(R):<br>**Adjuvant chemotherapy:**<br>Yes = 45<br>No = 48<br>Unknown = 5<br>**Chemoradiation:**<br>Neoadjuvant = 34<br>Adjuvant = 6<br>None = 57<br>Unknown = 1	98 (98)	Radiological (MRI/CT/PET-CT)	Yamada [8] and regional anatomical	(R):<br>Sphincter-preserving = 79<br>Non-sphincter-preserving = 19
Boyle K et al. 2005 [12]	UK	7	Retrospective	Y	(R):<br>56	64	(R):<br>38M : 26F	Dukes (R):<br>A = 8<br>B=19<br>C=26<br>Unknown = 11	Not reported	Not reported	64 (64)	Clinical/operative/radiological/histopathological	Regional anatomical (Leeds group) [21] and Symptoms & fixity (Mayo clinic) [7]	(R):<br>AR = 35<br>APER = 22<br>Proctectomy with end colostomy = 5<br>Total colectomy & end ileostomy = 1<br>Resection of rectal stump = 1
Brown W et al. 2017 [25]	Australia	4	Retrospective	Y	(R):<br>60	62	(R):<br>40M :22F	Not reported	Not reported	(R):<br>Radiotherapy alone = 9<br>Chemotherapy only = 2<br>Chemoradiotherapy = 30<br>None= 11<br>Unknown = 10	164 (164)	2 of the following: MRI/Pet-CT/CEA/clinical assessment	Anatomical	(R):<br>APER = 15<br>Non- APER = 47
Choi SH et al. 2018 [26]	Korea	7	Retrospective	N	Not reported	826	(TP):<br>500M : 326F	TNM (R):<br>T1-2/N0 = 9<br>T3N0 = 8<br>T3/N1 = 2<br>T3/N2 = 6<br>T4/N1 = 1<br>T4/N2 = 2	(R):<br>N0 = 17<br>N1 = 3<br>N2 = 8	No radiotherapy = 826	108 (31)	MRI	Anatomical	(R):<br>Curative TME = 28

Dresen R et al. 2010 [27]	The Netherlands	3	Retrospective	Y	(R):<br>61	40	(R):<br>26M : 14F	Not reported	Not reported	(R):<br>Neoadjuvant therapy:<br>No = 13<br>Short course RT = 17<br>Long course RT = 10	49 (49)	MRI	Anatomical	(R):<br>APER = 15<br>LAR = 25
Even-Sapir E et al. 2004 [28]	Israel	Not stated	Retrospective	N	(TP):<br>62	62	(TP):<br>37M : 25F	Not reported	Not reported	(TP):<br>Neoadjuvant chemoradiotherapy = 7<br>Adjuvant chemotherapy = 16<br>Adjuvant radiotherapy = 3	44 (44)	Clinical/radiological (CT,PET-CT,MRI,EUS)/endoscopy/histopathology	Anatomical	(TP):<br>17 = APER<br>45 = AR with pelvic anastomosis
Fujii S et al. 2009 [29]	Japan	20	Retrospective	Y	(R):<br>59	61	(R):<br>39M : 22F	Not reported	Not reported	Not reported	61 (61)	Not reported	Regional anatomical	Primary operative procedure not stated
Gilbertsen V et al. 1960 [69]	USA	10	Prospective	N	(R):<br>57.4	89	(R):<br>21M : 11F	Dukes (R):<br>A = 4<br>B = 4<br>C = 20<br>Unknown = 4	(R):<br>N-ve = 8<br>N+ve = 20<br>Unknown= 4	Not reported	39 (39)	Histopathological (Biopsy/surgical exploration/autopsy)	Anatomical	(R):<br>APER = 32
Gleeson F et al. 2012[30]	USA		Retrospective	N	(TP):<br>67	155	(TP):<br>98M : 57F	TNM (TP):<br>PT0 = 44<br>pT1 = 61<br>pT2 = 38<br>pT3 = 11<br>pT4 = 1	Not reported	(TP):<br>Neoadjuvant therapy = 6	16 (16)	Clinical/CEA/radiological (CT,MRI,EUS),histopathological	Anatomical	(R):<br>Transanal LE = 16
Hahnloser D et al. 2003 [31]	USA	15	Retrospective	Y	(R):<br>60.8	304	(R):<br>192M : 112F	Astler-Coller (R):<br>A=15<br>B1=54<br>B2=76<br>B3=15<br>C1=19<br>C2=72<br>C3=15<br>Unknown= 38	(R):<br>N-ve = 160<br>N+ve = 106<br>Unknown = 38	Not reported	304 (304)	CT/MRI/endoscopy/clinical	Symptoms & fixity (Mayo Clinic) [7]	(R):<br>Sphincter-preserving = 200<br>Stoma = 104
Hasegawa S et al. 2016 [32]	Japan	8	Retrospective	N	(TP):<br>64.8	212 (207 with previous R0 resection)	(TP):<br>145M : 67F	UICC (R):<br>I = 2<br>II = 16	(R) of 11 patients with LR other than pelvic sidewall nodes:<br><br>N-ve = 2<br>N+ve = 9	(TP):<br>**Neoadjuvant treatment:**<br>Chemotherapy = 30<br>Chemoradiotherapy = 7<br><br>In those with R0 resection:<br>**Adjuvant treatment:**<br>Chemotherapy = 81	19 (19)	Tumour markers/clinical examination/colonoscopy/ contrast enema/CT/MRI	Regional anatomical	(TP):<br>Sphincter-preserving = 178<br>Non-sphincter-preserving = 34<br>(PSD = 18, no PSD = 194)
Hruby G et al. 2003 [13]	New Zealand	13	Retrospective	Y	(R):<br>70	269	(R):<br>150M : 119F	TNM (R):<br>T1 = 4<br>T2 = 29<br>T3 = 198<br>T4 = 23<br>Unknown = 15	(R):<br>N-ve = 129<br>N+ve = 140	(R):<br>No radiotherapy = 269<br>Adjuvant chemotherapy = 36	268 (267)	(R):<br>Biopsy = 156<br>Combination of radiological/clinical findings = 113	Regional anatomical (Hruby) [31]	(R):<br>APER = 100<br>LE = 8<br>LAR = 154<br>Other = 6<br>Unknown = 1
Ingle P et al. 2019 [33]	India	3	Retrospective	Y	(R):<br>30	10	(R):<br>2M	TNM (R):<br>T3 = 2	(R):<br>N1 = 2	(R):<br>Neoadjuvant treatment:<br>Chemoradiotherapy then chemotherapy = 1<br>Short course radiotherapy = 1	2 (2)	Not reported	Anatomical	(R):<br>APER = 1<br>Intersphincteric resection = 1
Iversen H et al. 2017 [68]	Sweden	10	Retrospective	Y	(R):<br>65	95	(R):<br>59M : 36F	Not reported	Not reported	(R):<br>Neoadjuvant radiotherapy = 54	184 (184)	MRI	Regional anatomical (Memorial Sloan Kettering) [10]	Primary operative procedure not stated
Kanemitsu Y et al. 2010 [34]	Japan	25	Retrospective	Y	(R):<br>57	101	(R):<br>57M : 44F	Dukes (R):<br>A = 18<br>B = 21<br>C = 52<br>D = 5<br>Unknown = 5	(R) :<br>N-ve = 39<br>N+ve = 52<br>Unknown = 10	(R):<br>Adjuvant treatment:<br>Chemotherapy = 33<br>Radiotherapy = 3	101 (101)	CEA/PET CT/CT-guided biopsy/MRI<br><br>(MRI for location of pelvic recurrence)	Reginal anatomical Modified Yamada [8]	(R):<br>LE = 4<br>HAR = 15<br>LAR = 46<br>APR = 32<br>Hartmann's = 4
Killingback M et al. 2001 [35]	Australia	30	Prospective	N	(TP):<br>Male = 61.3<br>Female = 61.9	532	(TP):<br>304M : 228F	Dukes (R):<br>A = 5<br>B = 6<br>C = 30	(R):<br>N-ve = 11<br>N+ve = 30	(TP):<br>**Radiotherapy:** ^<br>Neoadjuvant = 28<br>Adjuvant = 5<br><br>Adjuvant chemotherapy = 1	Sphincter-saving resections: 49 (49)	Clinical examination & CT	Anatomical	(TP):<br>APER = 58<br>Sphincter-saving resection = 472<br>Proctocolectomy = 2<br><br>(R):<br>HAR = 1

														LAR = 6<br>Extended LAR = 5<br>AR with coloanal anastomosis =3<br>Unknown = 26
Kim T et al. 2008 [36]	Korea	4	Prospective	N	(TP):<br>57	366	(TP):<br>247M : 119F	TNM (TP):<br>cT3 = 354<br>cT4 = 12<br><br>ypT0 = 53<br>ypTis = 2<br>ypT1 = 19<br>ypT2 = 96<br>ypT3 = 182<br>ypT4 = 14<br><br>(R):<br>ypT<3 = 5<br>ypT≥3 = 19<br>Unknown = 5	(TP):<br>cN-ve = 58<br>cN +ve = 308<br><br>ypN0 = 250<br>ypN1 = 79<br>ypN2 = 37<br><br>(R):<br>ypN-ve = 8<br>ypN+ve = 16<br>Unknown = 5	(TP):<br>Neoadjuvant chemoradiotherapy = 366<br><br>Adjuvant chemotherapy = 357<br><br>(R):<br>Neoadjuvant chemotherapy = 24<br><br>Adjuvant chemotherapy = 23<br><br>Unknown = 5	30 (30)	Clinical/radiological (CT/MRI)l/histopathological	Regional anatomical	(TP):<br>Sphincter-preserving = 303<br>APER = 63<br><br>(R):<br>Sphincter-preserving = 18<br>APER = 6<br>Unknown = 5
Kim T et al. 2014 [37]	Korea	10	Prospective	N	Not reported	443	(TP):<br>292 M : 151 F	TNM (TP):<br>T1/2 = 26<br>T3/4 = 417	(TP):<br>N0 = 132<br>N+ve = 311	(TP):<br>Neoadjuvant chemoradiotherapy = 443<br><br>Adjuvant chemotherapy = 383	53 (53)	Biopsy/surgery/clinical imaging analysis (CT/PET-CT/MRI)	Regional anatomical	(TP):<br>APER = 77<br>LAR = 366
Kusters M et al. 2009 [38]	**Japan ***<br>& The Netherlands	9	Retrospective	N	(TP):<br>58	324	(TP):<br>215M : 109F	(TP):<br>pT1 = 52<br>pT2 = 107<br>pT3 = 160<br>pT4 = 5	(TP):<br>pN0 = 192<br>pN1 = 80<br>pN2 = 52	(TP):<br>Neoadjuvant therapy = 0<br><br>**Adjuvant therapy:**<br>Radiotherapy = 5<br>Chemotherapy = 23<br>None = 297	23 (21)	Clinical/radiological/histopathological	Regional anatomical (Kusters [39] and Roels [40])	(TP):<br>APER = 113<br>Hartmann's = 3<br>LAR = 195<br>PE = 13<br><br>LLND:<br>Standard TME = 134<br>Unilateral LLND = 69<br>Bilateral LLND = 121
Kusters M et al. 2009 [39]	The Netherlands	12	Prospective	N	(TP):<br>63	290 (247 with previous R0 resection)	(TP):<br>179M : 111F	(TP):<br>cT3+ = 113<br>cT4 = 177	Not reported	(TP):<br>**Neoadjuvant treatment:**<br><br>RT only = 86<br>Chemoradiotherapy = 204<br><br>IORT = 290<br><br>Adjuvant chemotherapy = 39	**Out of 247 patients with R0 resection:**<br><br>**18 (18)**<br><br>[34 LR in TP]	Clinical/radiological/histopathological	Regional anatomical (Kusters) [39]	(TP):<br>APER = 138<br>Abdominotranssacral resection = 12<br>LAR = 132<br>PE = 8
Kusters M et al. 2010 [40]	The Netherlands	Not stated	Prospective	N	(R):<br>65	1417	(R):<br>69M : 45F	TNM (R):<br>pT2 = 15<br>pT3 = 90<br>pT4 = 9	(R):<br>pN0 = 29<br>pN1 = 46<br>pN2 = 39	(R):<br>Neoadjuvant radiotherapy:<br>Yes = 36<br>No = 78	114 (114)	Clinical/radiological/histopathological	Regional anatomical (Kusters) [39]	(R):<br>APER = 47<br>Hartmann = 6<br>LAR = 61
Lambregts D et al. 2011 [41]	Netherlands	3	Retrospective	Y	(R):<br>68	42 (33 R0 resections)	(TP):<br>26M : 16F<br>(R):<br>13M : 6F	Not reported	Not reported	(TP):<br>Radiotherapy = 7<br>Neoadjuvant chemo+/- radiotherapy = 26<br><br>(R):<br>Radiotherapy = 2<br>Neoadjuvant chemo+/- radiotherapy = 11	19 (19)	MRI	Anatomical	(TP):<br>TME = 36<br>TEMS = 6<br><br>(R):<br>TME = 14<br>TEMS = 5
Luna-Perez P et al. 1999 [42]	Mexico	18	Prospective	N	(TP):<br>56.8	412<br><br>**Group 1:** 259<br><br>**Group 2:**<br>153	(TP):<br>230M : 182F<br><br>**Group 1:**<br>142M : 117F<br><br>**Group 2:**<br>88M : 65F	**Astler-Coller** and **Gunderson-Sosin** If tumour infiltrated into pelvic viscera<br>(TP):<br><br>**Group 1:**<br>No residual tumour = 12<br>A = 38<br>B1 = 37<br>B2 = 85	(TP):<br>**Group 1:**<br>N-ve =183<br>N+ve = 64<br>Unknown = 12<br><br>**Group 2:**<br>N-ve = 99<br>N+ve = 54	(TP):<br>Group 1 (neoadjuvant radiotherapy) = 259<br><br>(R):<br>Neoadjuvant RTx = 32	86 (85<br>(group 1 = 31, group 2 = 54)	CT	Anatomical	(TP):<br>Group 1:<br>APER = 149<br>LAR =. 74<br>PE = 36<br><br>Group 2:<br>APER = 62<br>LAR = 82<br>PE = 9

								B3 = 23<br>C1 = 23<br>C2 = 28<br>C3 = 13<br><br>**Group 2:**<br>A = 12<br>B1= 14<br>B2 = 66<br>B3 = 7<br>C1 = 5<br>C2 = 42<br>C3 = 7						
McDermott FT et al. 1985 [43]	Australia	33	Prospective	N	(TP):<br>Male= 59.1<br>Female = 60.9	1008	(TP):<br>552M : 456F	Astler-Coller (TP):<br>A = 276<br>B = 350<br>C1 = 285<br>C2 = 25<br>C unspecified = 7<br>D1 = 10<br>Unknown = 55	(TP):<br>N-ve = 626<br>N+ve = 317<br>Unknown =65	Not reported	320 (320)	Not reported	Anatomical	(TP):<br>Restorative resection = 644<br>Total excision = 364
Mendenhall W et al. 1983 [44]	USA	17	Retrospective	N	Not stated	90	Not stated	Astler-Coller (R):<br>B1=6<br>B2=12<br>C1=12<br>C2=11	(R):<br>N-ve = 18<br>N+ve = 23	(TP):<br>Surgery alone = 90	42 (41)	Clinical/radiological/histopathological	Anatomical	(TP):<br>APER = 54<br>AR = 28<br>Other = 7 (Hartmann's, partial colectomy, transverse colostomy, transrectal excision)<br><br>(R):<br>APER = 24<br>AR = 11<br>Other = 5<br>Unknown = 1
Messiou C et al. 2008 [45]	UK	6	Prospective	Y	(R):<br>57.1	49	(R):<br>28M : 21F	Not reported	Not reported	(R):<br>Perioperative radiotherapy = 17	75 (75)	MRI/ tumour markers/Biopsy (image-guided/colonoscopic/EUA)	Anatomical	(R):<br>APER = 19<br>AR = 25,<br>Excision of rectal stump = 1<br>Transanal excision = 4
Moore H et al. 2004 [9]	USA	6	Retrospective	Y	(TP):<br>59	119<br>(101 pelvic recurrence of rectal cancer, 18 pelvic recurrence of colon cancer)	(TP)<br>64M: 55F	TNM (TP): **<br>T0-2 = 37<br>T3-4 = 71	(TP): **<br>N0-X = 66<br>N1-2 = 46	**(TP):**<br>Adjuvant radiotherapy +/- chemotherapy = 59	(R):<br>In 101 pelvic recurrence of rectal cancer:<br><br>174 (174)	Retrospectively established by review of operative & histopathological reports	Regional anatomical (Memorial Sloan-Kettering) [10]	(R):<br>In 101 pelvic recurrence of rectal cancer:<br>APER = 15<br>LAR = 77<br>TAE = 8<br>Kraske = 1
Nagasaki T et al. 2014 [46]	Japan	8	Retrospective	Y	Not reported	30	(R):<br>Lap - 9M : 4F<br>Open - 12M : 5F	JSCCR (R):<br>Lap:<br>I = 2<br>II= 4<br>III= 6<br>IV=1<br><br>Open:<br>I = 4<br>II = 3<br>III = 7<br>IV= 3	(R):<br>Lap:<br>N-ve = 6<br>N+ve = 6<br>Unknown = 1<br><br>Open:<br>N-ve = 7<br>N+ve = 7<br>Unknown =3	(R):<br>Adjuvant chemotherapy<br>Lap = 6<br>Open = 9	30 (30)	Not reported	Anatomical	(R):<br>**Lap:**<br>LAR = 11<br>LAR with LLND = 1<br>Subtotal colectomy = 1<br>**Open:**<br>LAR = 14<br>APER = 2<br>Intersphincteric resection = 1
Novell F et al. 1997 [47]	Spain	7	Prospective	N	(TP):<br>67	140	(TP):<br>66M : 74 F	TNM (R):<br>T3-4, N0 = 5<br>T2-T4, N1 = 16	(R):<br>N0 = 5<br>N1 = 16	Not reported	21 (21)	(TP):<br>EUS & biopsy = 140<br>DRE= 14<br>Colonoscopy =16<br>CT Abdomen = 18<br>CEA = 13	Anatomical	(R):<br>AR = 21
Pergolizzi S et al. 1999 [48]	Italy	7	Retrospective	Y	(R):<br>64.5	39	(R):<br>24M : 15F	Dukes (R):<br>A = 13<br>B = 14<br>C = 12	(R):<br>N-ve = 27<br>N+ve = 12	(R):<br>No previous perioperative treatment = 39	39 (39)	Clinical/radiological/histopathological	Anatomical	(R):<br>19 = APER<br>20 =AR

Pilipshen S et al. 1984 [11]	USA	8	Prospective	N	(TP):<br>62	412	(TP):<br>243M : 169F	Dukes (R):<br>A = 18<br>B = 32<br>C = 55	(R):<br>N-ve = 50<br>N+ve = 55	(TP):<br>Neoadjuvant irradiation = 113<br>Adjuvant irradiation = 17<br><br>(R):<br>Neoadjuvant irradiation = 33	105 (105)	Clinical/biochemical/radiological/histopathological	Regional anatomical +/- fixation (Previous Memorial Sloan Kettering) [50]	(R):<br>APER = 39<br>LAR = 66
Rahbari N et al. 2011 [49]	Germany	7.5	Prospective	Y	(R):<br>62	92	(TP):<br>62M : 30F	UICC (R):<br>I = 17<br>II = 33<br>III = 35<br>IV = 7	(R):<br>N-ve = 50<br>N+ve = 35<br>Unknown = 7	(R):<br>Previous (chemo)radiotherapy = 51	92 (92)	Clinical/endoscopic/radiological/histopathological	Regional anatomical	(R):<br>Sphincter-preserving = 71<br>Non-sphincter-preserving = 21
Rich T et al. 1983 [50]	USA	4	Retrospective	N	Not reported	166 (142 with curative resection)***	(TP):<br>99M : 67 F	Dukes (R):<br>A = 3<br>B = 18<br>C = 22	(R):<br>N-ve = 21<br>N+ve = 22	(TP):<br>No previous perioperative treatment = 166	51 (51)	Clinical/endoscopic/radiological/histopathological	Anatomical	(TP):<br>CAPR = 110<br>LAR = 8<br>Hartmann's = 1<br>Abdominosacral resection = 3<br>(R):<br>CAPR = 34<br>LAR = 8<br>Hartmann's = 1
Roodbeen S et al. 2020 [51]	The Netherlands	7	Retrospective	N	(TP):<br>64	767	(R):<br>21M : 3F	(TP):<br>T1 = 23<br>T2 = 196<br>T3 = 421<br>T4 = 52<br>Tx = 4<br>Unknown = 71	(TP):<br>N0 = 214<br>N1 = 303<br>N2 = 175<br>Nx = 4<br>Unknown = 71	(R):<br>Previous neoadjuvant treatment = 17<br>Unknown = 7	24 (24)	Clinical/biochemical/radiological	Regional anatomical (Royal Marsden group) [11]	(TP):<br>TaTME:<br>APER/ELAPE = 91<br>Hartmann's = 5<br>LAR = 659<br>Proctocolectomy = 12
Safioleas M et al. 2005 [52]	Greece	13	Retrospective	N	(TP):<br>64	66 (52 had curative resection)	(TP):<br>29M : 37F	**Astler-Coller (R):**<br>B1 = 1<br>B2 = 4<br>C1 = 4<br>C2 = 3<br><br>**Dukes (R):**<br>B = 5<br>C = 7	(R):<br>N-ve = 5<br>N+ve = 7	(R):<br>Adjuvant chemoradiotherapy = 11	12 (12)	Radiological/histopathological	Anatomical	(R):<br>LAR = 12
Scialpi M et al. 1993 [53]	Italy	3	Prospective	N	(TP):<br>62.9	22	(TP):<br>12M: 10F	Astler-Coller (TP):<br>B1=4<br>B2=8<br>C1=1<br>C2=9 (The recurrences were both C2 pre-op)	(TP):<br>N-ve = 12<br>N+ve = 10<br><br>(R):<br>N+ve = 2	Not reported	2 (2)	TRUS/histopathological	Anatomical	(R):<br>LAR = 2
Sinaei M et al. 2013 [54]	Canada	11	Retrospective	Y	(R):<br>61	42	(R):<br>26M : 16F	Not stated	Not reported	Not reported	65 (65)	MRI	Regional anatomical (Memorial Sloan-Kettering) [10] and anatomical	(R):<br>APER = 16<br>Rectal anastomosis = 26
Suzuki K et al. 1996 [6]	USA	7	Prospective	Y	(R):<br>62.9	65	(R):<br>38M : 27F	Astler-Coller (R):<br>A = 6<br>B1 = 18<br>B2 = 11<br>B3 = 1<br>C1 = 4<br>C2 = 14<br>C3 = 3<br>Unknown = 8	(R):<br>N-ve = 36<br>N+ve = 21<br>Unknown = 8	Not reported	65 (65)	CT/CEA/biopsy	Symptoms and fixity (Mayo clinic) [7]	(R):<br>LAR = 34<br>APER = 15<br>Local excision = 15<br>Hartmann's = 1
Syk E et al. 2008 [55]	Sweden	9	Retrospective	N	(R):<br>73	2315	(TP):<br>1340M : 975F<br>(R):<br>89M : 66F	TNM (TP):<br>T1 = 575<br>T2 = 688<br>T3 = 719<br>T4 = 310<br>Unknown 23<br><br>TNM (R):<br>T1 = 14<br>T2 = 52<br>T3 = 70<br>T4 = 17<br>Unknown = 2	Not reported	**(TP):**<br>**Neoadjuvant treatment:**<br>RT 5x5 Gy + immediate surgery = 1155<br>RT 5x5 Gy + delayed surgery = 114<br>RT 25 x 2 Gy + surgery = 80<br>Chemoradiotherapy + surgery =32<br><br>No adjuvant radiotherapy = 2315	155 (124)	CT/MRI	Regional anatomical	(TP):<br>APER = 618<br>LAR = 1362<br>HAR (partial mesorectal excision) = 335<br>(R):<br>APER = 53<br>LAR = 72<br>HAR (partial mesorectal excision) = 30

										**(R):Neoadjuvant treatment :**<br>RT 5x5 Gy + immediate surgery = 49<br>RT 5x5 Gy + delayed surgery = 6<br>RT 25 x 2 Gy + surgery = 7<br>Chemoradiotherapy + surgery =3<br><br>No adjuvant radiotherapy = 155				
Uehara K et al. 2015 [56]	Japan	7	Retrospective	Y	(R):<br>66	35	(R):<br>27M : 8F	UICC (R):<br>I = 5<br>II = 15<br>III = 14<br>IV = 1	(R):<br>N-ve = 20<br>N+ve = 14<br>Unknown = 1	(R):<br>**Previous radiotherapy:**<br>For primary tumour = 2<br>For other disease = 1<br>None = 32	35 (35)	Not stated but CT/MRI/PET-CT for assessment of resectability	Regional anatomical (Hruby) [31]	(R):<br>Sphincter-preserving = 19<br>Non-sphincter-preserving = 16
Valentini V et al. 1999 [57]	Italy	8	Prospective	Y	(R):<br>62	47	(R):<br>29M : 18F	Not reported	Not reported	(R):<br>**External beam RT:**<br>Neoadjuvant = 7<br>Adjuvant = 6<br><br>Adjuvant chemotherapy = 6	47 (47)	CT/histopathological	Regional anatomical Modified Pilipshen (Memorial Sloan-Kettering) [50] and fixity (modified Mayo clinic) [7]	(R):<br>LAR = 33<br>APER = 14
Vigliotti A et al. 1987 [58]	USA	8	Retrospective	N	(TP):<br>56	105	(TP):<br>58M : 47F	Modified Astler-Coller (R):<br>B2=1<br>B3=4<br>C1=1<br>C2=8<br>C3=1	(R):<br>N-ve = 5<br>N+ve = 10	(R):<br>Adjuvant radiotherapy = 105	23 (23)	CT/histopathological	Anatomical in relation to radiotherapy field	(R):<br>AR = 6<br>CAPR = 9
Westberg K et al. 2017 [59]	Sweden	7	Retrospective	Y	(TP):<br>72	149 [$]	(R):<br>80M : 69F	Stage (R):<br>I = 26<br>II = 52<br>III = 68<br>Unknown = 3	(R):<br>N-ve = 78<br>N+ve = 68<br>Unknown = 3	(R):<br>Neoadjuvant treatment:<br>None = 93<br>Chemoradiotherapy = 56	149 (149)	Clinical/radiological/histopathological	Regional anatomical – combination of Leeds [21] and Memorial Sloan-Kettering [10]	(R):<br>APER = 26<br>Hartmann's = 16<br>LAR = 107
Wieldraaijer et al. 2018. [60]	The Netherlands	7	Retrospective	N	(TP):<br>65 [$$]	64* [$$]	(TP):<br>44M : 20F	Stage (TP):<br>pCR = 1<br>I = 9<br>II = 8,<br>III = 46	(TP):<br>N-ve = 18<br>Unknown = 46	Not reported [$$]	10 (10)	Clinical/radiological/histopathological/ endoscopic	Regional anatomical	(TP):<br>APER = 25<br>LAR = 39
Wiig JN et al. 1999 [61]	Norway	6	Prospective	Y	(R):<br>65	46	(R):<br>27M : 19F	Not reported	Not reported	Not reported	77 (77)	Clinical/radiological (CT)/endoscopic/histopathological	Regional anatomical	(R):<br>LAR = 46
Wong C et al. 1998 [62]	Canada	10	Retrospective	Y	(R):<br>55	519	(R):<br>290M : 229F	TNM (R):<br>T1N0 = 14<br>T2N0 = 59<br>T3-T4N0 = 173<br>T1-T2,N1-N2 = 14<br>T3-T4, N1-N2 = 201<br>Unknown = 58	(R):<br>N0 = 246<br>N1-N2 = 215<br>Unknown = 58	(R):<br>No previous adjuvant chemotherapy / irradiation = 519	519 (519)	Not stated. Histological confirmation = 261	Regional anatomical	(R):<br>APER = 326<br>LAR = 151<br>LE/electrocoagulation = 42
Yamada K et al. 2001 [7]	Japan	16	Retrospective	Y	Not reported	60	(R):<br>38M : 22F	Dukes (R):<br>A = 7<br>B = 16<br>C = 37	(R):<br>N-ve = 23<br>N+ve = 37	(R):<br>Most patients received adjuvant chemotherapy<br>Adjuvant radiotherapy = None	60 (60)	Radiological (CT/MRI/US/bone scintigraphy) and confirmed histopathologically	Regional anatomical (Yamada et. al) [8]	(R):<br>APER = 28<br>Sphincter-sparing = 32
You Y et al. 2012 [63]	USA	18	Retrospective	Y	(R):<br>57	46	(R):<br>22M : 24F	(R):<br>cT1 = 21<br>cT2 = 3<br>cT3 = 10<br>Unknown = 12<br><br>pT1 = 21<br>pT2 = 11<br>pT3 = 3<br>ypT2 = 7<br>ypT3 = 4	Not reported	(R):<br>Neoadjuvant chemoradiotherapy:<br>No = 35<br>Yes = 11<br><br>Adjuvant treatment: (chemotherapy/radiation/CRT) = 13	47 (47)	First mode of diagnosis:<br>Imaging = 20<br>Endoscopy = 13<br>CEA = 5<br>Pelvic pain = 5	Anatomical	(R):<br>Transanal LE = 46

Yu T et al. 2008 [64]	USA	12	Retrospective	N	(R):<br>55	554	(R):<br>14M : 22F	(R):<br>cT1-T2 = 4<br>cT3 = 21<br>cT4 = 7<br>Unknown = 4<br><br>pT1-T2 = 14<br>pT3 = 15<br>pT4 = 4<br>Unknown = 3	(R):<br>cN0 = 16<br>cN1-2 = 18<br>Unknown = 2<br><br>pN0 = 14<br>pN1-2 = 15<br>Unknown = 7	Not reported	46 (46)<br>36 patient images available (43)	(R):<br>CT = 36	According to radiotherapy field	(R):<br>APER = 9<br>LE = 8<br>LAR = 12<br>Pelvic exenteration = 2<br>Proctectomy/coloanal anastomosis = 4<br>Other = 1
Yun J et al. 2016 [65]	Korea	14	Retrospective	N	(R):<br>58	2050	(TP):<br>1233 M: 817F<br><br>(R):<br>84M : 63F	(R):<br>T0 = 2<br>T1 = 3<br>T2 = 22<br>T3 = 111<br>T4 = 9	(R):<br>N0 = 62<br>N1 = 44<br>N2 = 41	**(R):**<br>**Neoadjuvant treatment**<br>CCRT = 29<br><br>**Adjuvant chemotherapy**<br>Yes = 84<br>No = 34<br><br>**Adjuvant radiotherapy:**<br>Yes = 67<br>No = 51	147 (147)	Radiological (CT,PET-CT,MRI,EUS)/tumour markers/ histopathological	Regional anatomical (Kusters) [39]	(R):<br>TME - sphincter-preserving = 108<br>Non-sphincter-preserving = 39
Zhao J et al. 2012 [66]	Japan	8	Retrospective	N	(R):<br>59.4	1079	(R):<br>54M :36F	(R):<br>I = 6<br>IIA = 8<br>IIB = 11<br>IIIA = 10<br>IIIB = 20<br>IIIC = 26<br>Unknown = 9	(R):<br>N-ve = 25<br>N+ve = 56<br>Unknown = 9	(R):<br>Neoadjuvant chemoradiotherapy<br>Yes = 18<br>No = 72	90 (79)	CT/MRI	Regional anatomical (Memorial Sloan-Kettering) [10]	(R):<br>APER = 32<br>AR = 54<br>Hartmann's = 2<br>LE = 2
Zhu X et al. 2016 [67]	Japan	5	Retrospective	Y	(R):<br>56	135	(R):<br>73M : 62F	TNM (R):<br>T1 = 2<br>T2 = 35<br>T3 = 68<br>T4 = 30	(R):<br>N0 = 43<br>N1 = 62<br>N2 = 30	(R):<br>Radiotherapy:<br>Neoadjuvant = 19<br>Adjuvant = 6	135 (135)	Clinical/radiological (CT,PET-CT,MRI,EUS)/tumour markers/ histopathological	Regional anatomical based on Kusters [39] & Memorial Sloan-Kettering [10]	(R):<br>APER = 78<br>LAR = 57

Key: APR/APER = Abdominoperineal excision of rectum, AR = anterior resection, CAPR = Combined abdominoperineal resection, CCRT = concurrent chemoradiotherapy, EUS = Endorectal Ultrasound, HAR = High anterior resection, IORT = Intra-operative radiotherapy, is = in situ, JSCCR = Japanese Society for Cancer of the Colon and Rectum, Lap = laparoscopic, LAR = low anterior resection, LE = local excision, LLND = lateral lymph node dissection, pCR = pathological complete response, PE = pelvic exenteration, PSD = pelvic sidewall dissection, RT = radiotherapy, TAE = Transanal excision, TaTME = transanal TME, TME = total mesorectal excision, TNM = tumour, node, metastasis, TRUS = Transrectal ultrasound, UICC = Union for International Cancer Control, x = unassessable.

^ = None of these patients developed LR, * = Japanese patients only included here as Dutch TME trial patients already included above, ** = Data unavailable for all patients. Patients with R0 resection status only included, *** = The total population followed for recurrence, $ = including 27 patients who had R1 resection at primary surgery,
$$ = of the total number of patients with all recurrences. 378 patients in total, 64 with recurrence (local and distant) and 10 with LR.

Figure S1: Search strategy results

Search strategy 28th April 2020

MEDLINE (Pubmed)

(rectal cancer) AND (local recurrence) AND (patterns OR classification OR risk factors)

Studies identified: 1563

EMBASE (OvidSP)

1 (rectal cancer).af.
2 (local recurrence).af.
3 (patterns OR classification OR risk factors).af
4 1 AND 2 AND 3

Studies identified: 1091

Cochrane Central Register of Controlled Trials (CENTRAL)

#1 rectal cancer
#2 local recurrence
#3 patterns OR classification OR risk factors
#4 (#1 AND #2 AND #3)

Studies identified: 183
Trials: 114

Science Citation Index Expanded (via the Web of Science)

#1 TS=(rectal cancer)

#2 TS=(local recurrence)
#3 TS=(patterns OR classification OR risk factors)
#4 #1 AND #2 AND #3

Studies identified: 1140

All search results at Endnote: 3908

Duplicates: 1891

Search results after removal of duplicates: 2017

Studies for full-text: 229 [201 abstracts identified and papers located]

Studies identified through other sources: 29

Articles for full-text review: 230

Articles excluded after full-text review with reasons:

- No English version/translation of paper
- Conference abstracts
- No subclassification of LRRC only 'locally recurrent'
- Some anal/prostatic cancer papers

Final number of articles for inclusion in analysis: 58

Figure S2: Study Flow diagram

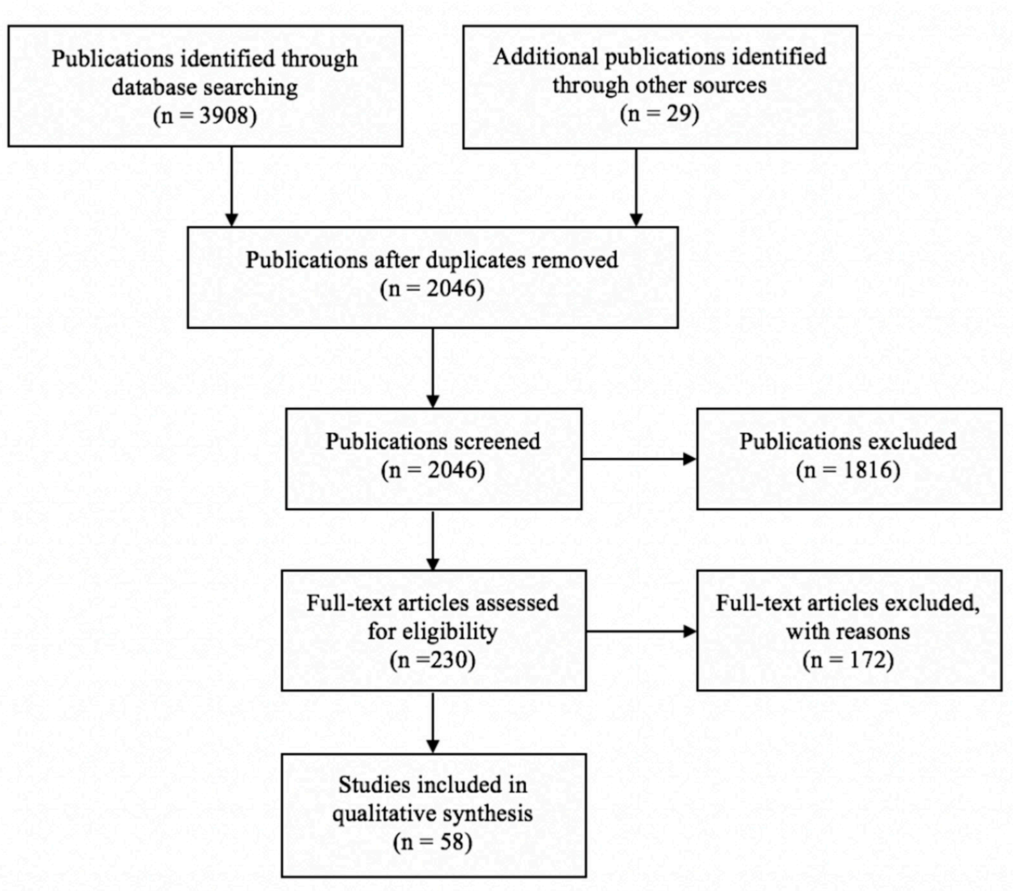

Supplement: Supplementary file 1 [file jcm-11-03511-s001.zip › jcm-1732082-supplementary.pdf]
